# DNA hypomethylation of a transcription factor binding site within the promoter of a gout risk gene *NRBP1* upregulates its expression by inhibition of TFAP2A binding

**DOI:** 10.1186/s13148-017-0401-z

**Published:** 2017-09-15

**Authors:** Zaihua Zhu, Weida Meng, Peiru Liu, Xiaoxia Zhu, Yun Liu, Hejian Zou

**Affiliations:** 1 Division of Rheumatology and Immunology, Huashan Hospital, Fudan University, Shanghai, China; 2 Institute of Rheumatology, Immunology and Allergy, Fudan University, Shanghai, China; 3 The Ministry of Education Key Laboratory of Metabolism and Molecular Medicine, Department of Biochemistry and Molecular Biology, School of Basic Medical Sciences, Fudan University, Shanghai, China

**Keywords:** Gout, Uric acid, DNA methylation, *NRBP1*, TFAP2A

## Abstract

**Background:**

Genome-wide association studies (GWASs) have identified dozens of loci associated with gout, but for most cases, the risk genes and the underlying molecular mechanisms contributing to these associations are unknown. This study sought to understand the molecular mechanism of a common genetic variant, rs780093, in the development of gout, both in vitro and in vivo.

**Results:**

Nuclear receptor binding protein 1 (*NRBP1*), as a gout risk gene, and its regulatory region, 72 bp upstream of the transcription start site, designated as B1, were identified through integrative analyses of genome-wide genotype and DNA methylation data. We observed elevated *NRBP1* expression in human peripheral blood mononuclear cells (PBMCs) from gout patients. In vitro luciferase reporter and protein pulldown assay results showed that DNA methylation could increase the binding of the transcription factor TFAP2A to B1, leading to suppressed gene expression. There results were further confirmed by in vivo bisulfite pyrosequencing showing that hypomethylation on B1 is associated with increased *NRBP1* expression in gout patients.

**Conclusions:**

Hypomethylation at the promoter region of *NRBP1* reduces the binding of TFAP2A and thus leads to elevated *NRBP1* expression, which might contribute to the development of gout.

**Electronic supplementary material:**

The online version of this article (10.1186/s13148-017-0401-z) contains supplementary material, which is available to authorized users.

## Background

Gout is a complex disorder caused by deposition of monosodium urate (MSU) crystals within joints with a prevalence of 1.14% in adults in China [1]. The male to female sex ratio is generally around 3–4 to 1 [2]. Initial symptom includes severely painful episodes of peripheral joint synovitis, but it can eventually lead to joint damage and deformity, chronic usage-related pain, and subcutaneous tophus deposition. Central to the development of gout is elevated serum uric acid concentrations [3]. Gout is also associated with many conditions that affect longevity and well-being, such as hypertension, diabetes mellitus, metabolic syndrome, and renal and cardiovascular disease [4–9]. In particular, gout is increasingly recognized as an independent cardiovascular risk factor [2, 10]. Over the past decade, genome-wide association studies (GWASs) and subsequent meta-analyses have led to a considerable expansion in the knowledge of common genetic loci that are associated with gout and elevated serum urate level [11–15]. However, apart from genes coding uric acid transporters, the mechanisms of how most of genetic variances identified from GWAS regulating urate level and developing into gout still remain poorly understood. As a common human disease, it is possible that both genetic predisposition and environmental exposure sharp its risk and contribute to its pathogenesis [16].

Epigenetics, such as histone modifications, DNA methylation, higher chromatin structure, and noncoding RNAs, can be influenced by genetic as well as environmental factors [17], and thus it can be a potential mechanism leading towards gout [18–20]. DNA methylation, an ancient and crucial epigenetic modification, is the heritable methylation of cytosines in the context of cytosine-guanine dinucleotides (CpGs) without changing the DNA sequence itself [21]. It is commonly believed that DNA methylation can disrupt the interactions between transcription factors (TFs) and DNA either directly [22] or indirectly by recruiting methyl CpG-binding proteins, such as MeCP2, that occupy the methylated promoters and compete for the TF binding sites [22]. However, some recent findings show that TFs could specifically bind to methylated CpG sites with specific DNA sequences (DNA motifs) and promote gene expression in vitro [23].

In the past decade, it has been shown that DNA methylation may play a role in many human autoimmune diseases, such as systemic lupus erythematosus, rheumatoid arthritis, multiple sclerosis, systemic scleroderma, and Sjogren’s syndrome [24]. The identification of dysregulation of DNA methylation may inspire the discovery of other uncharacterized mechanisms. Understanding the molecular mechanisms involved in the pathophysiology of autoimmune diseases is essential for the discovering of diagnostic biomarkers, as well as the introduction of effective and target-directed therapies [25]. However, there is no research regarding the role of epigenetics in gout up to now.

Here, by integrating genotype with DNA methylation, we identified a gout risk gene, nuclear receptor binding protein 1 (*NRBP1*). We demonstrated that hypomethylation of its promoter region, 72 bp upstream of *NRBP1* transcription start site (designated as B1 thereafter), is associated with increased gene expression both in vitro and in vivo. Moreover, gout-associated increased expression of *NRBP1* is regulated through methylation-dependent TFAP2A binding to the B1 region.

## Methods

### Study design

The study design is shown in Additional file 1: Figure S1.

### Clinical samples

The study was approved by the Ethics Committees of Huashan Hospital, Fudan University. PBMCs were harvested from males with gout before any medical treatment and matched healthy volunteers. Gout patients were diagnosed and met the requirements of the 1977 American Rheumatism Association preliminary criteria [26]. Gout patients were also with primary hyperuricemia, defined as serum uric acid concentrations higher than 7 mg/dL [27]. The inclusion criteria for gout patients are (1) no other diseases except for gout, (2) no treatments in the last 3 months, (3) male, (4) age between 18 and 50, and (5) never smoker. Clinical characteristics of cases and controls are shown in Table 1. Additionally, no study subjects have ever suffered from cancer. PBMCs from 17 gout patients and 15 matched controls were used for measurement of *NRBP1* RNA level and methylation of B1. PBMCs from an additional set of 7 gout patients and 7 matched controls were used for measurement of NRBP1 protein level.

**Table 1:** Clinical characteristics of study subjects

Characteristic	Cases (*n* = 15)	Controls (*n* = 17)	*P* value
Age (years)	39.33 ± 5.94	36.12 ± 7.66	0.12
Male (*n*)	15	17	1
Serum uric acid (μmol/L)	535.80 ± 38.31	320.65 ± 66.80	1.42E−11
Creatinine (μmol/L)	68.80 ± 28.18	56.94 ± 12.00	0.21
Body mass index (kg/m^2^)	24.81 ± 3.64	22.16 ± 3.52	0.04
Cholesterol (mg/dL)	197.20 ± 99.09	134.24 ± 49.02	0.03
Triglycerides (mg/dL)	149.73 ± 33.21	120.29 ± 43.33	0.04
Cancer (*n*)	0	0	1
Smoker (*n*)	0	0	1

Values are mean ± SD or numbers. *P* value for the difference between cases and controls

### Integrative analyses of genotype and DNA methylation

Data collection and processing for integrative genotype and DNA methylation data were performed as described previously with minor modifications [17]. Briefly, DNA methylation data from human blood and genotype data from the same cohort was obtained from the Gene Expression Omnibus (GEO) with accession number GSE42861. Disease-associated SNPs were collected from the NHGRI Catalog of Published GWASs [11–15], and a total of 105 SNPs, identified previously to be associated with gout or urate levels, were included for downstream genotype-methylation analyses. Associations between genotype and DNA methylation were evaluated using an additive minor-allele dosage model and SNP-CpG pairs with a stringent Bonferroni-adjusted *P* value less than 0.05 were determined to be significant, as suggested previously [17].

### Quantitative real-time PCR (qPCR)

Total cellular RNA of PBMCs was isolated with TRIzol reagent (Invitrogen, Carlsbad, USA). cDNA was generated by reverse transcription (PrimeScript RT Reagent kit; Takara), and the expression of *NRBP1* was quantified by qPCR using LightCycler 480 SYBR Green I Master Mix (Roche) normalized to *GAPDH* (more details in Additional file 2: File 1a). A non-template negative control (water) and a standard curve were conducted for each assay. The efficiency of amplification was determined based on the standard curve for mRNA quantification. All assays were performed in triplicates, and no obvious outlier was observed based on the SD.

### Generation of methylated DNA and luciferase reporter

Single-stranded DNA (ssDNA) were synthetized in Sangon Biotech (Shanghai, China) and annealed to generate double-stranded DNA (ds-DNA), which were then purified using Illustra MicroSpin G-25 Columns (GE Healthcare) according to the manufacturer’s protocol (Additional file 2: File 1b) [28]. Methylation of ds-DNA or luciferase reporter constructs was generated by their treatment with M.SssI CpG methyltransferase (Zymo Research) as described previously (Additional file 2: File 1b) [29]. The DNA were then recovered by ethanol precipitation. The methylation status was confirmed by bisulfite pyrosequencing.

### Protein pulldown assay

Biotinylated ssDNA were synthetized in Sangon Biotech (Shanghai, China) and annealed to generate dsDNA as described previously. Methylated or unmethylated biotinylated dsDNA probes with eight repeats of B1 were generated as above. GST-tagged human transcriptional factor TFAP2A was generated and purified from yeast as described previously [30]. The streptavidin magnetic beads (Roche, USA) were incubated with 1 pmol of the methylated or unmethylated biotinylated probes in the binding buffer (Additional file 2: File 1c) at room temperature for 10 min. The beads were then washed with washing buffer (Additional file 2: File 1c) for three times followed by incubation with 10 pmol of purified TFAP2A protein in PBS at 4 °C overnight. Beads were enriched on magnets and unbound supernatant were collected for Western blot. The beads, together with 10% input and 10% supernatant, were heated at 100 °C for 10 min and loaded on a 10% polyacrylamide gel by electrophoresis. The proteins were detected using antibody against TFAP2A (1:500; ab52222, Abcam) and visualized using luminescent image analyzer Image Quant Las 4000 mini (GE Healthcare Japan, Chiba, Japan).

### 293T cell culture

293T cells were maintained at 37 °C in 5% CO_2_ and grown in Dulbecco’s modified Eagle’s medium (GIBCO) supplemented with 10% fetal bovine serum (GIBCO). One day prior to transfection, 293T cells were seeded at a density of 100,000 cells per well on 48-well plates.

### Dual luciferase transcriptional reporter assay

The CpG-free luciferase reporter vector, pCpGL, is used for detecting promoter’s activity in dual luciferase transcriptional reporter assay [23]. Ds-DNA containing eight repeats of B1 (GGCGCAAGG) were synthesized and subcloned into pCpGL promoter region to generate pCpGL-8X-B1. Methylation of the luciferase reporter was carried out with M.SssI as described previously. The coding sequence (CDS) of TFAP2A was cloned into the expression vector, pCAGIG, with DNA recombination by using pEASY-Uni Seamless Cloning and Assembly Kit (Transgen, Shanghai, China). The following primers were used for the construct cloning; forwards: 5′-GTCTCATCATTTTGGCAAAGAATTCATCACAAGTTTGTACAAAAAAGCAG-3′ and reverse: 5′-ACGTAGCGGCCGCGATATCCTCGAGGCTTTCACCACTTTGTACAAGAAAG-3′. 293T cells were co-transfected with three constructs: pCpGL-8X-B1, pCAGIG expressing the TFAP2A at various concentrations, and pTK-RL (Promega, Madison, WI) using Lipofectamine 2000 (Life Technologies, USA). Cells were harvested 48 h post-transfection for luciferase reporter assay using the dual-luciferase reporter assay system (Promega), and signals were recorded by calculating firefly luciferase activity normalized to renilla activity. All assays were performed in triplicates and no obvious outlier was observed based on the SD.

### Sodium bisulfite pyrosequencing

Genomic DNA of PBMCs were extracted using the DNeasy Blood and Tissue Kit (Qiagen, Hilden, Germany). A total of 400 ng of genomic DNA were treated with sodium bisulfite using the EZ DNA Methylation-Gold Kit (Zymo Research). Twenty-five nanograms of bisulfite-converted genomic DNA was amplified with unbiased nested PCR reaction using ExTaq DNA Polymerase (Takara, Kyoto, Japan). The primer sequences to amplify B1 region at the promoter of *NRBP1* were as follows: outer primers forward: 5′-TTATTATTGAATGATAATTTTAATGAGTT-3′ and reverse: 5′-CCTAAACTACTAAATAAACAAAACC-3′; and inner primers forward: 5′-GTAGAATTATTTGGGGTATTTGGAT-3′ and reverse: 5′ biotin-AACCCTCTTTTCCCTAAAC-3′. To quantify the percentage of methylated cytosine in each CpG site, amplified DNA were sequenced using a pyrosequencing system (PyroMark Q96, Qiagen) [31]. This method treats each individual CpG site as a C/T polymorphism and generates quantitative data for the relative proportion of the methylated versus the unmethylated allele. The sequencing primer 5′-GGTGGGGTGGATAGAGA-3′ was designed as reverse run, and the percentage of DNA methylation on each measured CpG site was generated by Pyro Q-CpG methylation software (Biotage). A non-template negative control (water) and a standard curve were included for each run.

### Statistical analysis

All results were expressed as means ± SEM of at least three independent experiments. Comparisons between groups were evaluated by using an unpaired two-tailed Student’s *t* test. The association between DNA methylation and gene expression was calculated by a linear regression model. A *P* value less than 0.05 was considered statistically significant.

## Results

### Identification of the gout risk gene *NRBP1*, by integrative analyses of GWAS with DNA methylation data

Our previous work has showed that DNA methylation can be influenced by genotype and integrative genotype-methylation analyses can help us to fine map the epigenetic variants that might be responsible for the disease phenotype [17]. Given this, we want to investigate how altered DNA methylation level may play a role in disease etiology, specifically in gout. To do this, we first examined the genotype-methylation associations for 105 SNPs, identified previously to be associated with gout or urate level, from the NHGRI Catalog of Published GWASs [11–15]. We identified six disease-associated SNPs that were significantly associated with altered DNA methylation level (Table 2). Among them, only one CpG site, cg05102552, is located in the promoter region of a gene. Since it is well known that promoter methylation can regulate the gene expression, we thus decided to focus on this region for the downstream study.

**Table 2:** CpG sites whose methylation are controlled by gout-associated GWAS SNPs

GWAS SNP	CpG	Chromosome	SNP position (hg19)	CpG position (hg19)	Gene close to the CpG site	*P* value (geno vs. meth)
rs780093	cg05102552	chr2	27742603	27650867	*NRBP1*	3.13E−12
rs13129697	cg00071950	chr4	9926967	10020882	*SLC2A9*	3.87E−12
rs3775948	cg00071950	chr4	9997112	10020882	*SLC2A9*	< 2E−16
rs717615	cg00071950	chr4	10104670	10020882	*SLC2A9*	< 2E−16
rs1165205	cg15691649	chr6	25870542	25882328	*SLC17A3*	< 2E−16
rs505802	cg19131476	chr11	64357072	64387923	*NRXN2*	< 2E−16

**Fig. 1:**
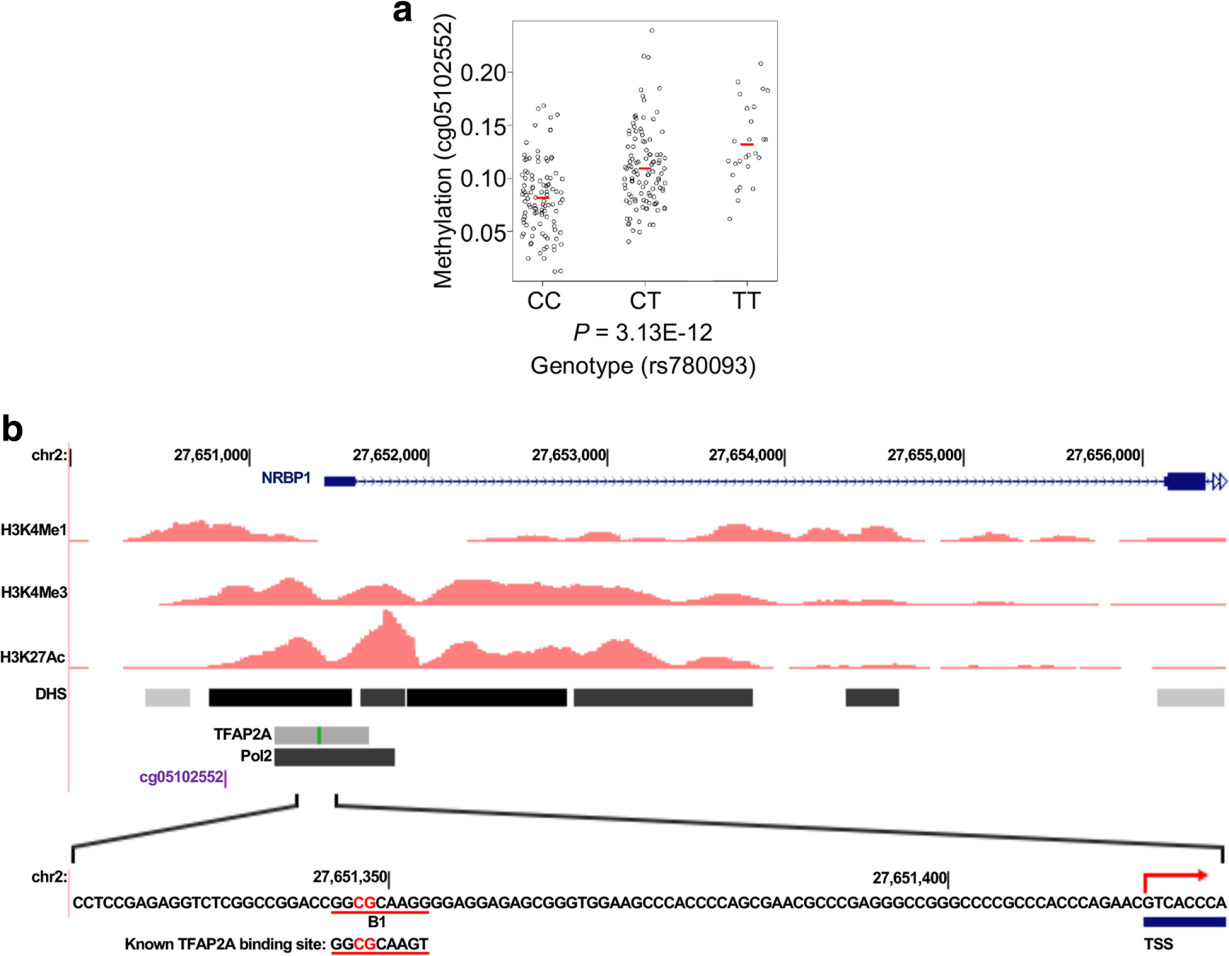
Identification of a gout risk gene and a potential TFAP2A binding site at its promoter region. **a** The association between a gout-associated GWAS SNP, rs780093, and DNA methylation level of cg05102552 from human peripheral blood mononuclear cells (PBMCs). Each dot represents an individual and average methylation level for each genotype group is indicated by red bar. Statistical significance for the association was evaluated with additive minor-allele dosage model and *P* value was indicated in the bottom. **b** Cg05102552 site is 552 bp upstream of *NRBP1* transcription start site (TSS) and is in a region with DNase I hypersensitive sites (DHSs) and high level of active histone modifications, such as H3K4me1, H3K4me3, and H3K27Ac. The potential TFAP2A binding region, B1, was highlighted in the bottom

**Fig. 2:**
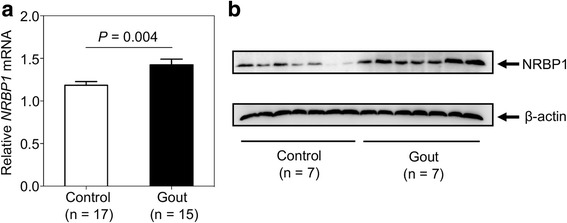
NRBP1 is overexpressed in PBMCs from gout patients. **a** Relative *NRBP1* mRNA expression in freshly isolated PBMCs from 15 gout patients and 17 healthy controls detected by quantitative real-time PCR (qPCR). The expression level of *NRBP1* is normalized to *GAPDH* and is represented as mean ± SEM. (*P* value = 0.004, Student’s *t* test, unpaired, two-sided). **b** NRBP1 protein expression in freshly isolated PMBCs from 7 gout patients and healthy controls detected by Western blotting

The methylation level of cg05102552 is strongly associated with a SNP, rs780093 (Fig. 1a), which has been shown to associate with gout in both Caucasian [12] and Han Chinese populations [32]. Cg05102552 site is 552 bp upstream of *NRBP1* transcription start site and is in a region with DNase I hypersensitive sites (DHSs) [33, 34] and high level of active histone modifications, such as H3K4me1, H3K4me3, and H3K27Ac (Fig. 1b) [35], suggesting that it may be the regulatory region for *NRBP1* expression. Furthermore, both normalized RNA level (gout versus control 1.425 ± 0.0656 versus 1.185 ± 0.0432 (mean ± SEM); *P* value = 0.004) and protein level of *NRBP1* in PBMCs were found to be significantly elevated in gout patients (Fig. 2), indicating that *NRBP1* may be a gout risk gene.

### Identification of a potential TFAP2A binding site at the promoter region of *NRBP1*

We next investigated how methylation at the promoter region of *NRBP1* may regulate its gene expression. We overlapped this region with the Encyclopedia of DNA Elements (ENCODE) database and observed that, in addition to epigenetic marks associated with accessible chromatin, this region is also occupied by transcription factors, such as TFAP2A (Fig. 1b) [36]. Moreover, the DNA sequence in this region contains a motif highly similar to a known TFAP2A binding site (Fig. 1b), identified previously with protein microarrays in vitro [23]. We thereafter designated this potential TFAP2A binding site, 72 bp upstream of *NRBP1* transcription start site, as B1.

### *NRBP1* expression is regulated by methylation-dependent TFAP2A binding to its promoter region in vitro

**Fig. 3:**
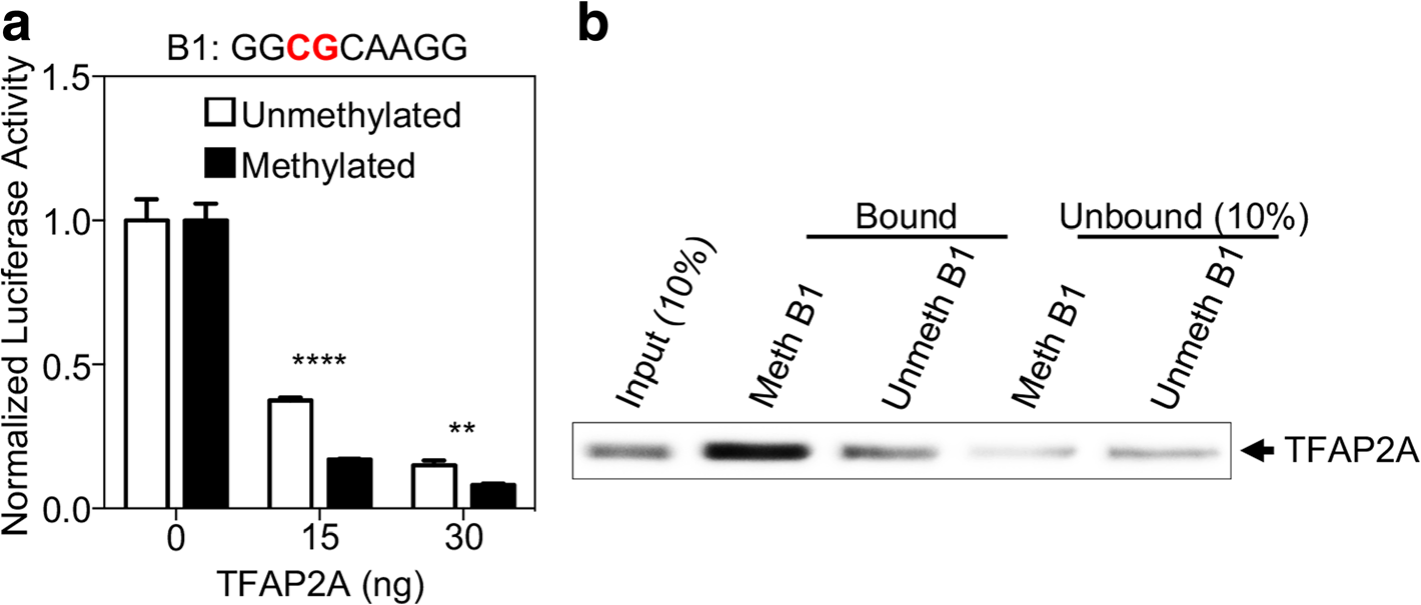
Increased DNA-binding of TFAP2A to methylated B1 inhibits gene transcription in vitro. **a** TFAP2A exhibited methylation-dependent inhibition of luciferase activity on B1. Dual-luciferase reporter assays were performed to evaluate the transcriptional activity of the methylated or unmethylated B1 by TFAP2A in 293T cells, with firefly luciferase activity normalized to renilla activity. Data are represented as mean ± SEM (**P value < 0.001, ****P value < 0.00001, Student’s *t* test, unpaired, two-sided). **b** DNA methylation on B1 can increase its direct interaction with TFAP2A. Purified TFAP2A was incubated with streptavidin bead-bound, biotinylated B1 with or without DNA methylation modification. Bead-bound proteins were fractionated by SDS-PAGE, and TFAP2A was detected by immunoblotting. Portions (10%) of the purified TFAP2A (input) or unbound fractions were assayed in parallel

To determine whether B1 is a potential TFAP2A binding site important for *NRBP1* expression, we performed a cellular-based dual-luciferase reporter assay. We observed significant reduction of reporter gene expression in the presence of TFAP2A, and this reduction was TFAP2A dose dependent (Fig. 3a), indicating that B1 is indeed a TFAP2A regulatory motif for gene expression. Moreover, the reduction of gene expression was much more enhanced after we methylated B1 region on the luciferase reporter (Fig. 3a), suggesting that B1 can regulate gene expression in a methylation-dependent manner in the presence of TFAP2A. To further interrogate whether this dose- and methylation-dependent inhibition of gene expression is mediated through direct TFAP2A binding to the B1 motif, we conducted a pulldown experiment and observed that purified TFAP2A protein could specifically bind to the B1 motif and this binding was much stronger if the CpG dinucleotide within B1 was methylated (Fig. 3b). Taken together, these results suggest that the transcription factor, TFAP2A, can specifically bind to the B1 region at the promoter of *NRBP1* and inhibit gene expression in vitro, and DNA methylation on B1 can further enhance the binding of TFAP2A and the inhibition of gene expression.

### Hypomethylation of B1 is associated with increased *NRBP1* expression in gout patients

**Fig. 4:**
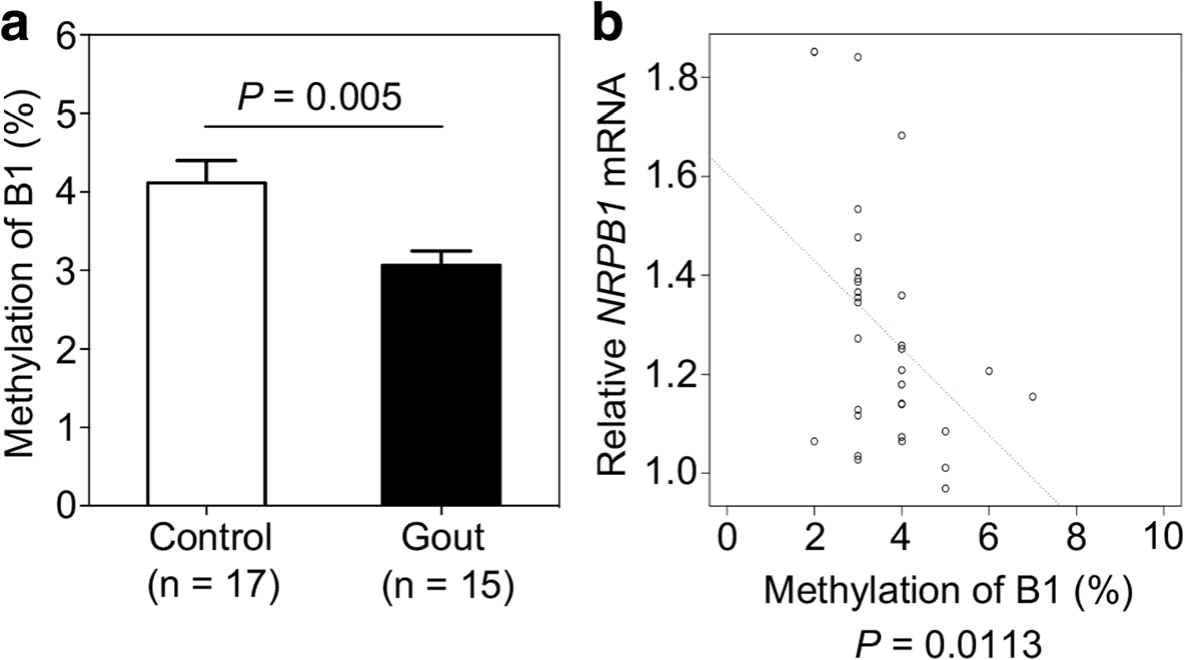
Hypomethylation of B1 is associated with increased *NRBP1* expression in gout patients. **a** Bisulfite pyrosequencing showed DNA methylation on B1 at the promoter of *NRBP1* is significantly lower in gout patients (*n* = 15) comparing to healthy controls (*n* = 17). Data are represented as mean ± SEM. (*P* value = 0.005, Student’s *t* test, unpaired, two-sided). **b** A significant negative association between *NRBP1* gene expression and DNA methylation on B1 in the tested subjects (*P* value = 0.0113)

In order to test that increased *NRBP1* expression we observed in gout patients is regulated through methylation-dependent TFAP2A binding to *NRBP1* promoter region, we evaluated the DNA methylation level of the CpG dinucleotide on B1 using PBMCs of gout patients (*n* = 15) and healthy controls (*n* = 17). We observed that DNA methylation on B1 was significantly lower from gout patients compared to healthy controls (gout versus control 3.067 ± 0.182% versus 4.118 ± 0.2829%; *P* value = 0.005) (Fig. 4a). Moreover, there is a significant negative association between *NRBP1* gene expression and DNA methylation on B1 (*P* value = 0.011) (Fig. 4b), which is consistent with our in vitro findings that hypomethylation on B1 is associated with increased gene expression. In addition to gout disease status, we also observed a marginal significant association between DNA methylation of B1 and serum uric acid (*P* value = 0.08) (Additional file 1: Figure S2a) and a significant association between *NRBP1* expression and serum uric acid (*P* value = 0.03) (Additional file 1: Figure S2b). Thus, these results support the idea that hypomethylation of B1 leads to increased *NRBP1* expression in gout patients.

## Discussion

**Fig. 5:**
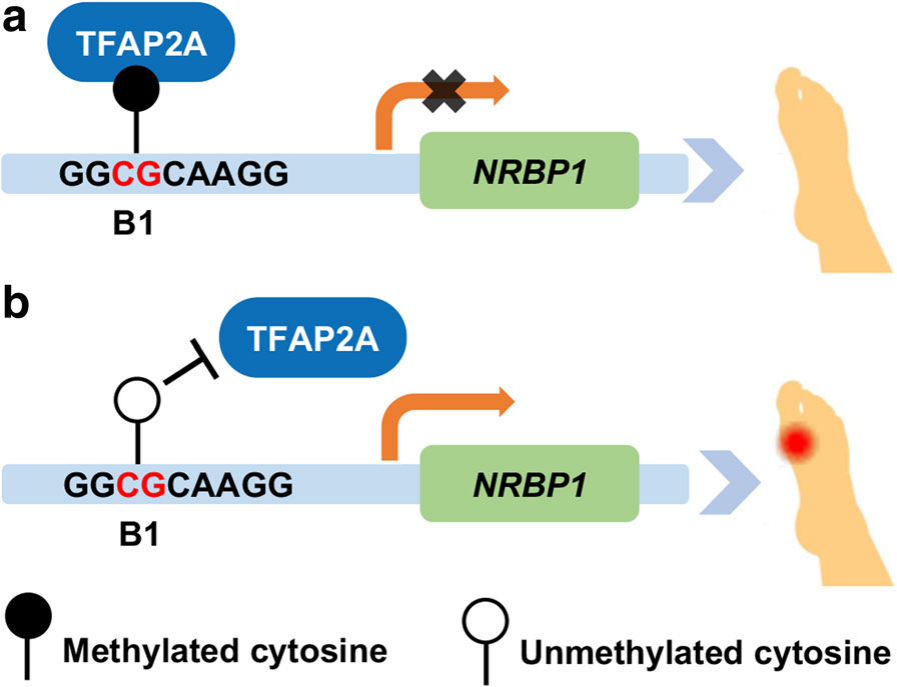
Proposed model for methylation-dependent TFAP2A binding and regulation of *NRBP1* expression in gout. **a** In healthy subjects, TFAP2A binds to methylated B1, which suppresses the transcription of *NRBP1*. **b** In contrast, hypomethylation of B1 at the promoter region of *NRBP1* abrogates TFAP2A binding, which results in elevated *NRBP1* expression in gout

In summary, we have identified a gout risk gene, *NRBP1*, using integrative analyses of genotype and DNA methylation. Through experiments both in vitro and in vivo, we demonstrated that increased *NRBP1* expression in gout patients may be regulated through methylation-dependent binding of TFAP2A to the B1 region, 72 bp upstream of *NRBP1* transcription start site (Fig. 5).

Our data showed that even though TFAP2A can bind to unmethylated B1 (Fig. 3b) and inhibit gene expression (Fig. 3a) when B1 is not methylated, the binding between DNA and TFAP2A was much enhanced when B1 is methylated, and this leads to greater reduction of gene expression. However, since we only investigated the role of B1 methylation in vitro, whether hypomethylation of B1 can lead to reduced gene expression in vivo is still unknown. The direct functionality of cg05102552 methylation and its role in regulating *NRBP1* gene expression still needs to be investigated in the future.

In our current work, we have identified and shown that *NRBP1* may be a risk gene in gout. However, the understanding of downstream pathways explaining detailed molecular mechanisms awaits further study. *NRBP1*, previously described to play a role in tumor suppression, cellular homoeostasis, and protein regulation [37], was recently reported to be associated with an autoimmune disease, Takayasu’s arteritis [38]. Studies have shown that *NRBP1* can regulate the Wnt/β-catenin signaling pathway [39, 40] and affect the expression of ATP binding cassette subfamily G member 2 (*ABCG2*) [41], a critical urate efflux transporter in kidney proximal tubule and intestine [42]. Considering the important role of *ABCG2* in gout, it is worth further investigating the molecular mechanism of *NRBP1* in gout development.

Even though we showed that there is a statistically significant difference for DNA methylation of B1 between gout patients and healthy controls, the effect size for the difference is small. This is actually not uncommon to observe relatively small methylation difference associated with human common diseases [43–45]. Moreover, methylation level for the CpG sites around the B1 region is also decreased in gout patients (Additional file 1: Figure S3), suggesting that this is a differentially methylated region (DMR). Considering methylation level in the neighboring CpGs is usually correlated [17], this provides an additional evidence supporting hypomethylation of B1 in gout. Additionally, since DNA methylation level analyzed in current study was from PBMCs, which may not be the directly relevant tissue for gout etiology, methylation difference may be much severer in tissues directly involved in gout, such as leucocytes and monocytes/macrophages in affected joints or tissues in kidney and intestine that excrete uric acid. Nevertheless, methylation difference in blood can still reflect the epigenetic regulation involving in the disease mechanism and may be used as a potential biomarker for early diagnosis or intervention before gout attack and severe complication [24].

In spite of these limitations above, to the best of our knowledge, this is the first report showing how DNA methylation may affect the pathogenesis of gout. Contradictory to the conventional wisdom that methylated CpG dinucleotide at the promoter can abrogate transcription factor binding and lead to transcriptional silencing, we demonstrated that DNA methylation can also inhibit gene expression by facilitating transcription factor binding to the promoter. Given DNA methylation is reversible, this work provides a promising target for potential gout therapy.

## Conclusions

In conclusion, we identified a gout risk gene, *NRBP1,* by integrating genome-wide genotype with DNA methylation data. We demonstrated that hypomethylation of its promoter region, B1, 72 bp upstream of *NRBP1* transcription start site, is associated with increased gene expression both in vitro and in vivo. Moreover, gout-associated increased *NRBP1* expression is regulated through methylation-dependent TFAP2A binding to the B1 region, which might be involved in the pathogenesis of gout.

## Additional files

Additional file 1: Figure S1. Work flow diagram. **Figure S2.** Serum uric acid is regulated by B1 methylation level and *NRBP1* expression. **a** A marginal significant negative association between DNA methylation of B1 and serum uric acid (*P* value = 0.08). **b** A significant positive association between *NRBP1* expression and serum uric acid (*P* value = 0.03). **Figure S3.** Decreased DNA methylation at the promoter region of *NRBP1* in gout patients. **a** The DNA sequence at the promoter region of *NRBP1* gene. The CpG sites, designated as B1 to B6, are highlighted in red. **b** The methylation level for each CpG site indicated in **a**, was investigated by bisulfite pyrosequencing. Data are represented as mean ± SEM. (*P value <0.01, **P value <0.001, Student’s *t* test, unpaired, two-sided). (PPTX 1077 kb)

Additional file 2: File 1. Detailed experimental methods. **a** Quantitative real-time PCR. **b** Generation of methylated DNA and luciferase reporter. **c** Protein pulldown assay. (DOCX 13 kb)

## Abbreviations

*ABCG2*: ATP binding cassette subfamily G member 2
CpGs: Cytosine-guanine dinucleotides
DHSs: DNase I hypersensitive sites
DMR: Differentially methylated region
ENCODE: Encyclopedia of DNA Elements
GEO: Gene Expression Omnibus
GWASs: Genome-wide association studies
MSU: Monosodium urate
*NRBP1*: Nuclear receptor binding protein 1
PBMCs: Peripheral blood mononuclear cells
TF: Transcription factor
