# Supplementary material for: DNA hypomethylation of a transcription factor binding site within the promoter of a gout risk gene NRBP1 upregulates its expression by inhibition of TFAP2A binding
Source: Clin Epigenetics. 2017 Sep 15;9:99. doi: 10.1186/s13148-017-0401-z (PMC5603049; doi:10.1186/s13148-017-0401-z)
Supplement: Supplementary file 1 — Work flow diagram. Figure S2. Serum uric acid is regulated by B1 methylation level and NRBP1 expression. a A marginal significant negative association between DNA methylation of B1 and serum uric acid (P value = 0.08). b A significant positive association between NRBP1 expression and serum uric acid (P value = 0.03). Figure S3. Decreased DNA methylation at the promoter region of NRBP1 in gout patients. a The DNA sequence at the promoter region of NRBP1 gene. The CpG sites, designated as B1 to B6, are highlighted in red. b The methylation level for each CpG site indicated in a, was investigated by bisulfite pyrosequencing. Data are represented as mean ± SEM. (*P value <0.01, **P value <0.001, Student’s t test, unpaired, two-sided). (PPTX 1077 kb) [file 13148_2017_401_MOESM1_ESM.pptx]

## Slide 1
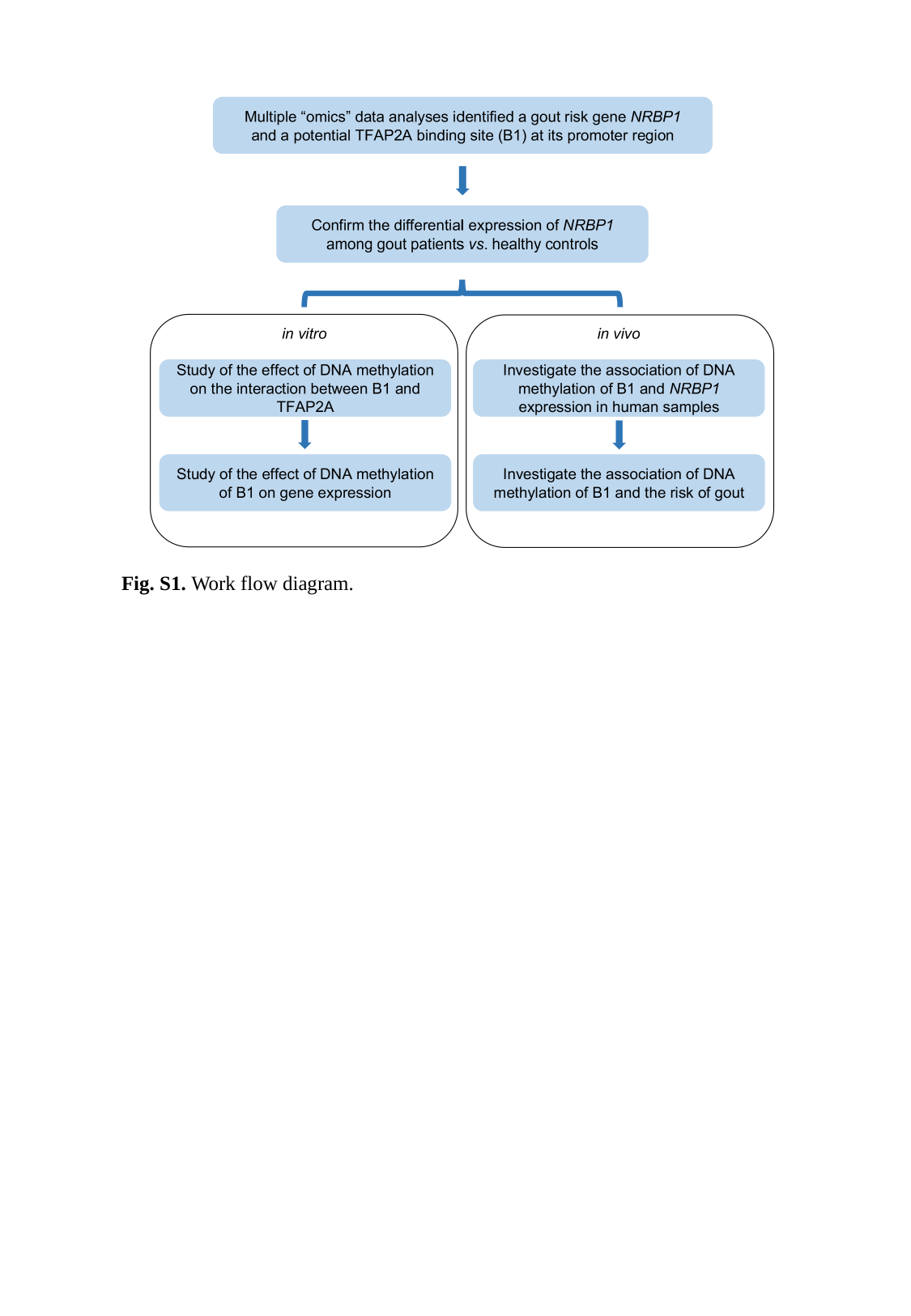

Fig. S1. Work flow diagram.

## Slide 2
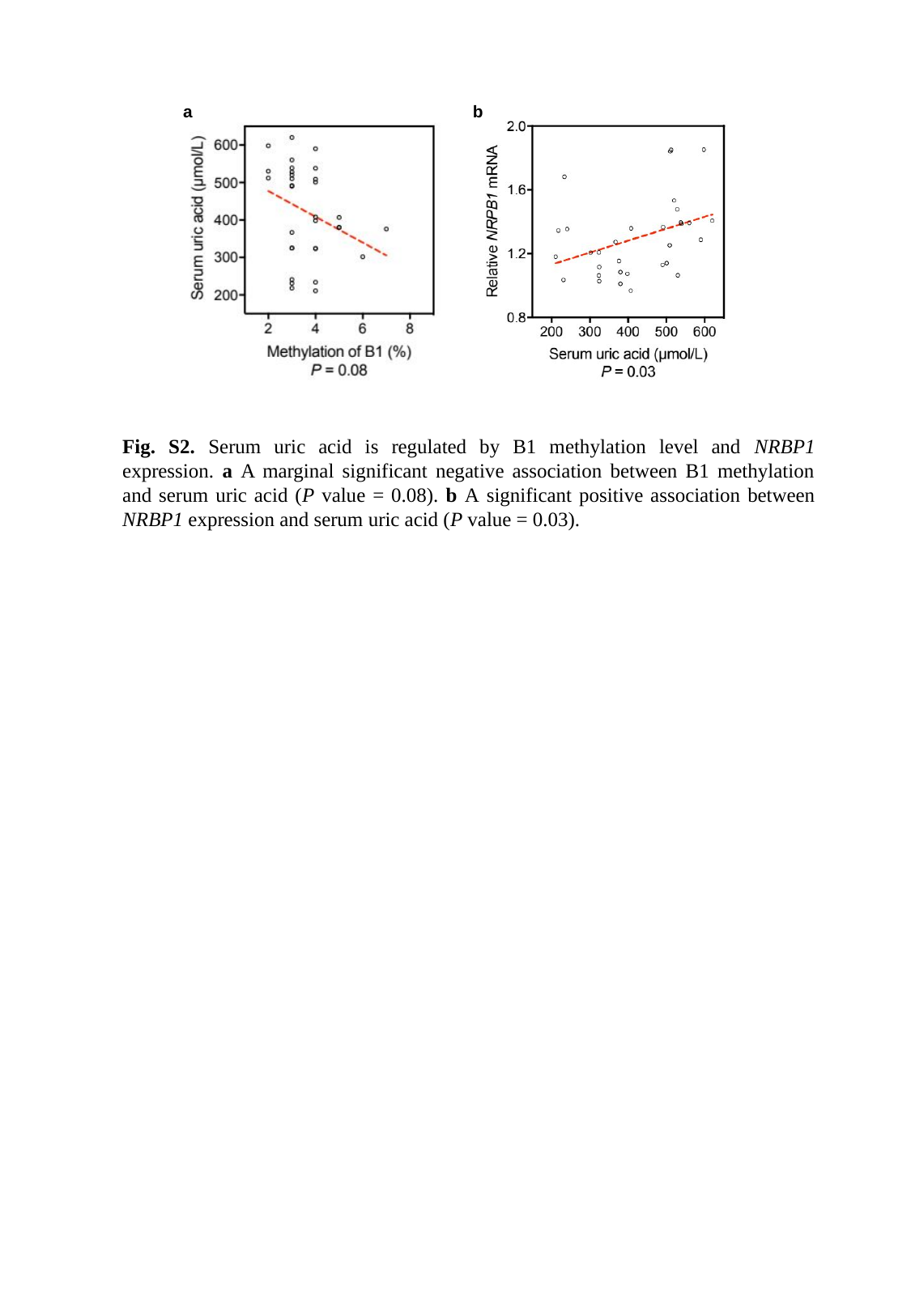

a
b
Fig. S2. Serum uric acid is regulated by B1 methylation level and NRBP1 expression. a A marginal significant negative association between B1 methylation and serum uric acid (P value = 0.08). b A significant positive association between NRBP1 expression and serum uric acid (P value = 0.03).

## Slide 3
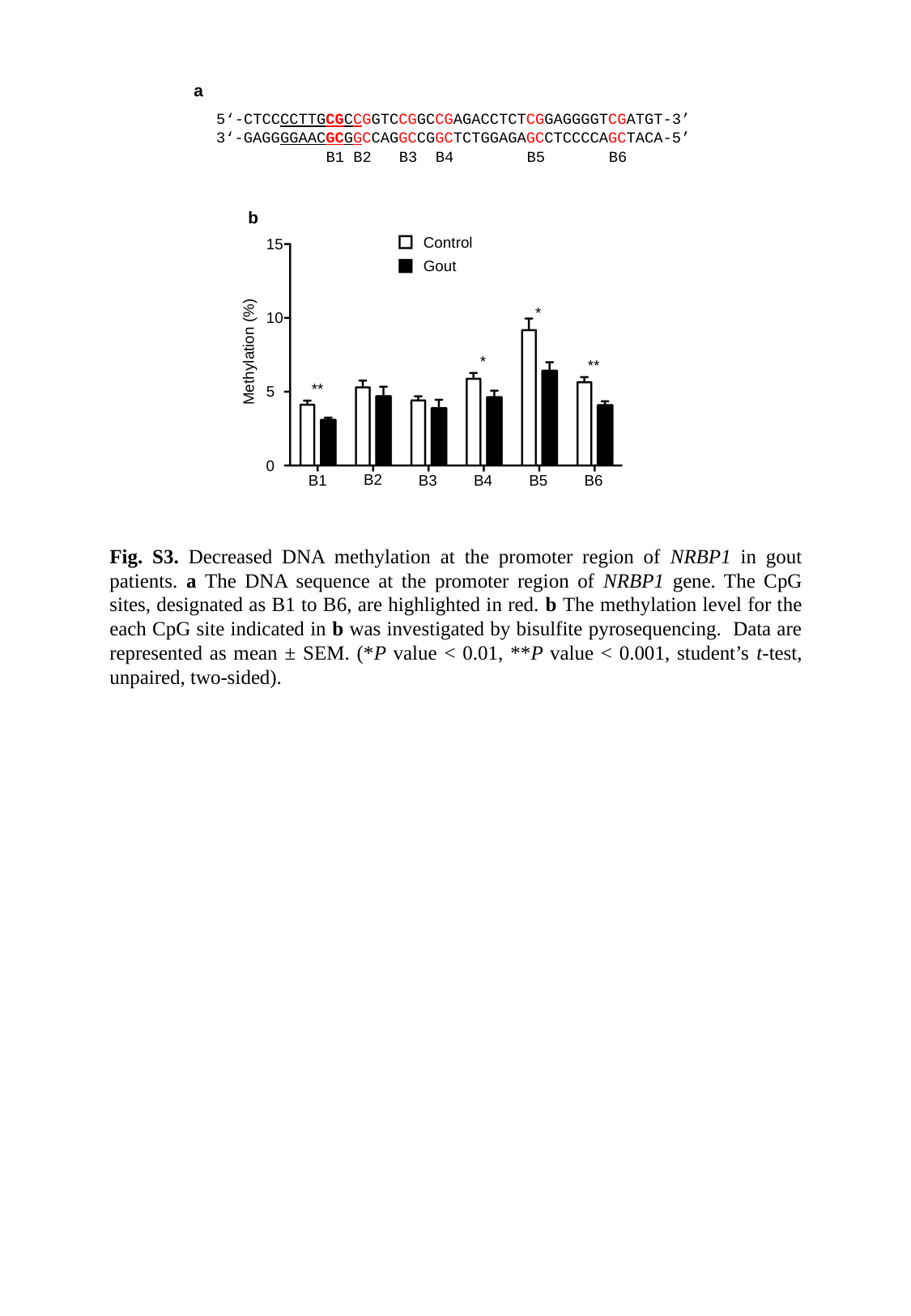

a
5‘-CTCCCCTTGCGCCGGTCCGGCCGAGACCTCTCGGAGGGGTCGATGT-3’
3‘-GAGGGGAACGCGGCCAGGCCGGCTCTGGAGAGCCTCCCCAGCTACA-5’
B1 B2 B3 B4 B5 B6
b
Control
15
Gout
*
10
Methylation (%)
*
**
**
5
0
B2
B3
B4
B5
B6
B1
Fig. S3. Decreased DNA methylation at the promoter region of NRBP1 in gout patients. a The DNA sequence at the promoter region of NRBP1 gene. The CpG sites, designated as B1 to B6, are highlighted in red. b The methylation level for the each CpG site indicated in b was investigated by bisulfite pyrosequencing. Data are represented as mean ± SEM. (*P value < 0.01, **P value < 0.001, student’s t-test, unpaired, two-sided).
