# Supplementary material for: DNA hypomethylation of a transcription factor binding site within the promoter of a gout risk gene NRBP1 upregulates its expression by inhibition of TFAP2A binding
Source: Clin Epigenetics. 2017 Sep 15;9:99. doi: 10.1186/s13148-017-0401-z (PMC5603049; doi:10.1186/s13148-017-0401-z)
Supplement: Supplementary file 2 — Detailed experimental methods. a Quantitative real-time PCR. b Generation of methylated DNA and luciferase reporter. c Protein pulldown assay. (DOCX 13 kb) [file 13148_2017_401_MOESM2_ESM.docx]

**Additional file 1**

**File 1a. Quantitative real-time PCR (qPCR)**

*NRBP1* mRNA was quantified by qPCR with primers, forward: 5’-AGATGGCAGTGCTGGAGATT-3’ (chr2: 27,660,127-27,660,146) and reverse :5’-AGGGCACTTCAAACAATGCT-3’ (chr2: 27,662,704-27,662,723). The PCR cycling conditions are as follows: 95°C for 5 minutes, followed by 45 cycles of 95°C for 10 seconds, 60°C for 10 seconds, and 72°C for 10 seconds. Relative *NRBP1* gene expression was calculated by normalizing its level to that of GAPDH. A non-template negative control and a standard curve were conducted for each assay. All assays were performed in triplicates and outliers were excluded by checking the SD.

**File 1b. Generation of methylated DNA and luciferase reporter**

Oligonucleotide stock solutions of 100 μM were prepared using molecular biology grade Tris-EDTA buffer solution. Further dilutions were carried out in annealing buffer containing 10 mM Tris-HCl (pH 7.5), 50 mM sodium chloride and 1 mM EDTA. Samples were annealed by heating at 98 °C for 10 minutes followed by slow cooling to room temperature at a controlled rate of 0.1 °C/min to generate double-stranded DNA (ds-DNA). Ds-DNA were then purified using Illustra MicroSpin G-25 Columns (GE Healthcare). Methylation of ds-DNA or luciferase reporter constructs was generated by their treatment with M.SssI CpG methyltransferase (Zymo Research). Briefly, 0.4 μg of DNA were incubated with 0.7 μl of M.SssI CpG methyltransferase (4 U/μl, Zymo Research), 0.7 μl of S-adenosylmethionine (SAM, 12 mM) and 2 μl of 10×CpG Reaction Buffer in a 20 μl reaction volume at 30°C for 2 hours. Then 0.7 μl of M.SssI (4 U/μl) and 0.7 μl of SAM (12 mM) were added into the reaction and incubated at 30°C overnight, followed by additional 0.6 μl of M.SssI (4 U/μl) and 0.6 μl of SAM (12 mM) the next morning with incubation at 30°C for 2 hours. The reaction was terminated by incubation at 65°C for 15 minutes.

**File 1c.** **Protein pulldown assay**

Binding buffer: 10 mM Tris-HCl (pH 7.5), 1 mM EDTA and 100 mM NaCl

Washing buffer: 10 mM Tris-HCl (pH 7.5), 1 mM EDTA and 1 M NaCl
